# Genome-wide association study of nevirapine hypersensitivity in a sub-Saharan African HIV-infected population

**DOI:** 10.1093/jac/dkw545

**Published:** 2017-01-05

**Authors:** Daniel F. Carr, Stephane Bourgeois, Mas Chaponda, Louise Y. Takeshita, Andrew P. Morris, Elena M. Cornejo Castro, Ana Alfirevic, Andrew R. Jones, Daniel J. Rigden, Sam Haldenby, Saye Khoo, David G. Lalloo, Robert S. Heyderman, Collet Dandara, Elizabeth Kampira, Joep J. van Oosterhout, Francis Ssali, Paula Munderi, Giuseppe Novelli, Paola Borgiani, Matthew R. Nelson, Arthur Holden, Panos Deloukas, Munir Pirmohamed

**Affiliations:** 1 Department of Molecular and Clinical Pharmacology, University of Liverpool, Liverpool, UK; 2 William Harvey Research Institute, Barts and The London School of Medicine and Dentistry, Queen Mary University of London, London, UK; 3 Malawi-Liverpool-Wellcome Trust Clinical Research Programme, College of Medicine, University of Malawi, Malawi; 4 Institute of Integrative Biology, University of Liverpool, Liverpool, UK; 5 Department of Biostatistics, University of Liverpool, Liverpool, UK; 6 Centre for Genomic Research, University of Liverpool, Liverpool, UK; 7 Liverpool School of Tropical Medicine, Liverpool, UK; 8 Division of Infection and Immunity, University College London, London, UK; 9 Division of Human Genetics, University of Cape Town, Cape Town, South Africa; 10 Dignitas International, Zomba, Malawi; 11 Joint Clinical Research Centre, Headquarters, Kampala, Uganda; 12 UVRI/MRC Uganda Research Unit on AIDS, Entebbe, Uganda; 13 Department of Biomedicine and Prevention, University of Rome ‘Tor Vergata’, Rome, Italy; 14 GlaxoSmithKline, Research Triangle Park, NC, USA; 15 SAEC Consortium, Ltd, Chicago, IL, USA; 16 Wellcome Trust Sanger Institute, Hinxton, Cambridge, UK; 17 Princess Al-Jawhara Al-Brahim Centre of Excellence in Research of Hereditary Disorders (PACER-HD), King Abdulaziz University, Jeddah, 21589, Saudi Arabia

## Abstract

**Background:** The antiretroviral nevirapine is associated with hypersensitivity reactions in 6%–10% of patients, including hepatotoxicity, maculopapular exanthema, Stevens–Johnson syndrome (SJS) and toxic epidermal necrolysis (TEN).

**Objectives:** To undertake a genome-wide association study (GWAS) to identify genetic predisposing factors for the different clinical phenotypes associated with nevirapine hypersensitivity.

**Methods:** A GWAS was undertaken in a discovery cohort of 151 nevirapine-hypersensitive and 182 tolerant, HIV-infected Malawian adults. Replication of signals was determined in a cohort of 116 cases and 68 controls obtained from Malawi, Uganda and Mozambique. Interaction with *ERAP* genes was determined in patients positive for HLA-C*04:01. *In silico* docking studies were also performed for HLA-C*04:01.

**Results:** Fifteen SNPs demonstrated nominal significance (*P *<* *1 × 10^−5^) with one or more of the hypersensitivity phenotypes. The most promising signal was seen in SJS/TEN, where rs5010528 (*HLA-C* locus) approached genome-wide significance (*P *<* *8.5 × 10^−8^) and was below *HLA*-wide significance (*P *<* *2.5 × 10^−4^) in the meta-analysis of discovery and replication cohorts [OR 4.84 (95% CI 2.71–8.61)]. rs5010528 is a strong proxy for HLA-C*04:01 carriage: *in silico* docking showed that two residues (33 and 123) in the B pocket were the most likely nevirapine interactors. There was no interaction between HLA-C*04:01 and *ERAP1*, but there is a potential protective effect with *ERAP2* [*P *=* *0.019, OR 0.43 (95% CI 0.21–0.87)].

**Conclusions:** HLA-C*04:01 predisposes to nevirapine-induced SJS/TEN in sub-Saharan Africans, but not to other hypersensitivity phenotypes. This is likely to be mediated via binding to the B pocket of the HLA-C peptide. Whether this risk is modulated by *ERAP2* variants requires further study.

## Introduction

Nevirapine, an NNRTI used for HIV^1^ infection is effective^2^ as part of combination antiretroviral therapy, but causes hypersensitivity in 6%–10% of patients.^3^^,^^4^ This can manifest in various ways, ranging from nevirapine-induced rash (NIR) (i.e. a maculopapular exanthema without any systemic manifestations), hypersensitivity syndrome (HSS) to severe blistering skin reactions such as Stevens–Johnson syndrome (SJS) and toxic epidermal necrolysis (TEN)^5^ (1–2 per 1000 exposed individuals^6^). Extra-cutaneous involvement typically manifests as hepatotoxicity.^7^

Identification of the genetic risk factors for nevirapine hypersensitivity has focused on candidate gene approaches. Nevirapine is primarily metabolized by the hepatic cytochrome P450s 2B6 (CYP2B6) and 3A4 (CYP3A4).^8^ The exon 4 variant in *CYP2B6* (c.516G > T), which encodes a non-synonymous amino acid substitution (Gln172His) (rs3745274), leads to loss of function,^9^^,^^10^ with the variant T allele resulting in higher nevirapine plasma concentrations in both Caucasian^11^ and sub-Saharan^12^ adult patients. The associations with *CYP2B6* polymorphisms are rather confusing with the *CYP2B6* c.516G > T SNP associated with nevirapine-induced cutaneous adverse events in black and white populations^13^ but not with nevirapine-induced hepatotoxicity.^14^ The association with HLA alleles is even more complex, with HLA-DRB1*01:01 (Caucasian^13^^,^^15^^,^^16^), HLA-C*04 (Thai,^17^ Chinese^18^ and Black^13^), HLA-C*08 (Japanese^19^) and HLA-B*35:05 (Thai^13^^,^^20^) acting as predisposing alleles for nevirapine hypersensitivity. Our own previous study within a subset of patients from the Malawian HIV population described in this paper identified an association between HLA-C*04:01 and nevirapine-induced SJS.^21^

In this study, in order to overcome some of the issues associated with candidate gene analysis, we have undertaken a genome-wide association study (GWAS) in a Malawian HIV cohort of nevirapine-exposed patients in order to identify genetic biomarkers of nevirapine hypersensitivity in an unbiased manner. We have also investigated whether there is any interaction between HLA-C*04:01 in SJS/TEN patients and the endoplasmic reticulum aminopeptidase genes (*ERAP1* and *ERAP2*), which have been shown to modulate the risk of various immune diseases, in particular ankylosing spondylitis.^22^

## Methods

### Patients

#### Discovery cohort

**Figure 1:**
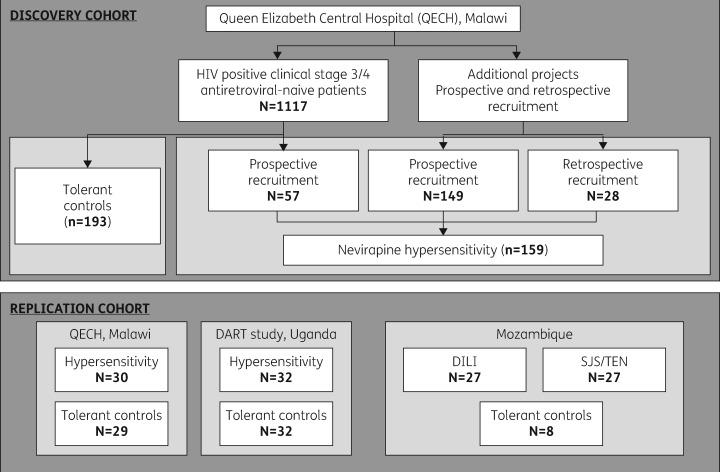
Schematic representation of the source of both nevirapine-hypersensitive and -tolerant patients for the GWAS discovery and replication cohorts.

Antiretroviral-naive patients (*n *=* *1117) were prospectively recruited as previously described^21^ (Figure 1) from the Queen Elizabeth Central Hospital (QECH), Blantyre, Malawi, between March 2007 and December 2008. All were self-reported black African, over the age of 16, and had no baseline jaundice. CD4+ counts and liver function tests were monitored at 0, 2, 6, 10, 14, 18 and 22 weeks. Fifty-seven patients from this prospective cohort had nevirapine-induced hypersensitivity fulfilling the criteria of one or more of the following phenotypes:

- NIR: widespread maculopapular exanthema with no systemic manifestations but which worsened on treatment continuation.
- HSS: widespread rash with systemic manifestations (i.e. fever, cough or abnormal liver function tests). This is also known as DRESS (drug reaction with eosinophilia and systemic symptoms).
- SJS: blistering eruption affecting <10% of body surface area with two or more mucous membranes involved.
- TEN: blistering rash affecting >30% of body surface area and two or more mucous membranes. Patients with overlap syndrome had 10%–30% of their body surface area affected.
- Drug-induced liver injury (DILI): jaundice and abnormal ALT.

In addition, a total of 149 cases of nevirapine-induced hypersensitivity were recruited prospectively from QECH separately from the study described above, and a further 28 were identified retrospectively from patient records at the same centre. Out of a total of 234 hypersensitive cases, 159 where sufficient genomic DNA was available were included, along with 193/1060 of the nevirapine-treated age- and gender-matched controls (352 in total), in the discovery GWAS. Numbers of tolerant controls included were constrained by DNA quality and quantity.

#### Replication cohort

We recruited a number of patients with nevirapine hypersensitivity, with different phenotypes, from a number of centres (Table 1) to replicate our findings:

**Table 1:** Non-genetic data for nevirapine-tolerant and -hypersensitive patient cohorts included in both the main and replication analyses after sample exclusion based on genotyping QC criteria

	Cases [median (range)]					Controls [median (range)]					Phenotype (*n*)			
	*n*	age	female	BMI	CD4+	*n*	age	female	BMI	CD4+	NIR	HSS	SJS/TEN	DILI
Discovery cohort^a^	151	35 (17–69)	63%	20.5 (14.6–41.4)	235 (3–906)	182	35 (17–63)	60%	20.2 (13.7–36.4)	170 (2–677)	56	23	51	21
Replication cohort														
Malawi^b^	30	36 (24–58)	48%	19.8 (13.1–26.8)	207 (15–560)	29	36 (24–56)	47%	19.9 (14.9–30.3)	145 (25–314)	13	3	9^c^	6^c^
Uganda	32	37 (24–62)	73%	21.1 (8.9–39.6)	73 (17–201)	31	37 (25–53)	71%	21.3 (11.5–35.6)	66 (11–196)	14	8	2	10
Mozambique (SJS/TEN)^d^	27	31 (21–52)	100%	22.8 (12.4–35.0)	467 (107–906)	8	31 (23–41)	100%	27.5 (26.1–35.0)	556 (244–682)	–	–	27	–
Mozambique (DILI)	27	32 (23–43)	100%	23.7 (13.7–34.0)	324 (23–2305)	–	–	–	–	–	–	–	–	27
combined	116	34 (21–62)	78%	21.7 (8.9–39.6)	198 (15–2305)	68	36 (23–56)	64%	20.9 (11.5–35.6)	102 (11–682)	27	10	38	42

a CD4+ data missing for six cases.

b CD4+ data missing for four hypersensitive patients and BMI data missing for one hypersensitive patient.

c One patient presented with SJS/TEN and DILI phenotype.

d CD4+ data missing for three hypersensitive and four tolerant patients, and BMI data missing for six hypersensitive and four tolerant patients.

- Thirty nevirapine-hypersensitive patients and matched (age and gender) HIV-positive nevirapine-treated controls from Malawi. All controls and eight of the cases were from the original study but not included in the initial GWAS due to DNA quantity restraints. The other 22 cases presenting with the hypersensitivity phenotype according to the above criteria were identified from the QECH after the conclusion of the initial recruitment phase (December 2008).
- Thirty-two nevirapine-hypersensitive cases and age- and gender-matched controls identified in Uganda from the DART study cohort.^23^ Cases were defined according to available patient records and subsequently categorized into the sub-phenotypes described above.
- Twenty-seven pregnant female patients with nevirapine-induced hepatotoxicity and 10 nevirapine-tolerant pregnant controls from Mozambique. Cases were defined as previously stated,^24^ and included patients who discontinued nevirapine due to increased liver enzymes (grade 3/4). Controls were excluded if ALT/AST levels exceeded the median value observed in the case cohort.
- Twenty-seven female patients with nevirapine-induced SJS/TEN from Mozambique.^25^ In this instance SJS/TEN was defined as development of exanthema and blistering starting mainly on the trunk, involving ≥10% of the body surface with mucosal involvement.

### Ethics

Full ethics approval for the study was received from the Liverpool School of Tropical Medicine Research Ethics Committee (Liverpool, UK), the College of Medicine Research and Ethics Committee, University of Malawi (Blantyre, Malawi) and the Uganda National Council for Science and Technology. All patients gave their written informed consent and those who met the criteria for a case had nevirapine withdrawn in accordance with Malawian National Treatment Guidelines. Local ethics approval was obtained for the DART study as previously described^23^ with subsequent ethics approval for a pharmacogenetic sub-study also obtained.^26^

### DNA extraction

Genomic DNA was extracted from whole blood for the discovery cohort^21^ and replication cohorts as previously described.^24^^,^^25^

### Discovery cohort genotyping and sample quality control (QC)

A total of 352 samples were genotyped for 1 048 713 variants using the HumanOmni1-Quad_v1 chip (Illumina). Variants were excluded from analysis if their minor allele frequency (MAF) was <1%, the call rate was <99% for an MAF between 1% and 3%, the call rate was <98% for an MAF of >3%, or if Hardy–Weinberg expectations were not satisfied (*P *<* *10^−^^4^).

Individuals were excluded if the sample call rate was <95%, the assigned gender contradicted genetic information from the X chromosome heterozygosity, or if they appeared to be duplicates, or related to other individuals in the study (as measured by identity by state using PLINK^27^). Multidimensional scaling analysis of genotype data was undertaken by merging the data with HapMap3 cohort data and using the mds function in PLINK^27^ in order to determine population stratification (Figure S1, available as Supplementary data at *JAC* Online).

### Discovery cohort imputation

Imputation of genotypes, after phasing of each chromosome using ShapeIt,^28^ was carried out using IMPUTE V2.3.1,^29^ 1000G phase 1 integrated v3 macGT1 reference panel haplotypes (March 2012).^30^ After imputation, SNPs with an information measure (info score) <0.8 were discarded, and a threshold of 0.5 was applied on genotype uncertainty. Imputed variants with an MAF <1% were then excluded.

### Discovery cohort association analysis

Univariate logistic regression analysis of non-genetic covariates (age, gender, BMI, CD4+ cell count) was undertaken for each hypersensitivity phenotype. Statistically significant variables (*P *<* *0.05) were included in the subsequent logistic regressions to test for the association of each hypersensitivity phenotype with each SNP passing QC. All statistical analyses were undertaken using PLINK^27^ and R.^31^ Given prior associations between nevirapine hypersensitivity and *HLA* allele associations, it was felt reasonable to specify a Bonferroni-corrected *HLA*-wide significance threshold of *P *<* *2.5 × 10^−^^4^, based on the presumption that there are usually <200 effective HLA allele tests.

### Replication cohort genotyping, QC and association analysis

SNPs determined to have a nominally significant association with a nevirapine hypersensitivity phenotype (*P *<* *1 × 10^−^^5^) in the discovery cohort were subsequently typed in the replication cohort using either the Sequenom MassArray iPLEX platform (Sequenom Inc., San Diego, CA, USA) or custom TaqMan real-time PCR SNP genotyping assays (Life Technologies, Paisley, UK) according to the manufacturer’s protocols. SNPs were excluded if they failed to meet the genotype QC thresholds as outlined for the discovery cohort or if assay design software parameters prohibited their inclusion.

Logistic regression analysis of the replication cohort, including and excluding CD4+ count as a covariate, where appropriate (as determined in the discovery cohort), was carried out. Meta-analysis of combined discovery and replication cohorts was undertaken using a fixed-effects model with inverse-variant effect size weighting in GWAMA.^32^

### Imputation of HLA allelotype and MHC locus

Imputation of *HLA-C* allelotype from the discovery cohort SNP array data was undertaken using HLA*IMP:02^33^ (see Supplementary data).

### HLA-C and ERAP gene–gene interactions

In cases and tolerant controls positive for carriage of the rs5010528 G allele, which was used as a proxy for HLA-C*04:01, we investigated both *ERAP1* (rs10050860 and rs30187) and *ERAP2* (rs2248374, rs2549782) SNPs (using data from the Illumina array), which have previously been shown to interact with HLA-mediated immune diseases. Association of *ERAP1* and *ERAP2* SNPs with SJS/TEN risk was determined in the HLA-C*04:01-positive cohort (cases and controls) by logistic regression with CD4+ cell count as a covariate using PLINK.^27^ A Bonferroni adjustment for multiple testing was applied with a significance threshold of *P *=* *0.125.

### Targeted sequencing of MHC region

Sixteen genomic DNA samples from nevirapine-induced SJS/TEN and 16 age- and gender-matched tolerant controls were carried forward for MHC-targeted sequencing. The methodology is detailed in the Supplementary data.

### Allelotyping

*HLA* allelotyping was performed from raw FASTQ data files using Omixon Target v1.81 HLA Typing software and utilized the HLA database version 3.15.0. (Omixon Ltd, Budapest, Hungary).

### In silico docking

In order to predict possible modes of interaction between nevirapine and HLA-C*04:01, *in silico* docking was undertaken. The methodology is detailed in the Supplementary data.

## Results

### Discovery cohort

A total of 333 samples (151 cases and 182 controls) out of 352 passed QC. Of the 19 excluded samples, 9 failed heterozygosity checks (outliers by >3 SD), 8 failed identity checks and 2 failed the call rate threshold. Multi-dimensional analysis for population stratification (Figure S1) demonstrated no population outliers. In total, 817 728 SNPs passed QC and were carried over for imputation with the 1000 genomes panel. Imputation produced a dataset of 1 421 8511 variants. Cohort characteristics are shown in Table 1. We considered five nevirapine-induced hypersensitivity phenotypes for analysis—NIR, HSS, SJS/TEN, DILI (Table 1)—and also combined these different phenotypes into an overall hypersensitivity group.

**Figure 2:**
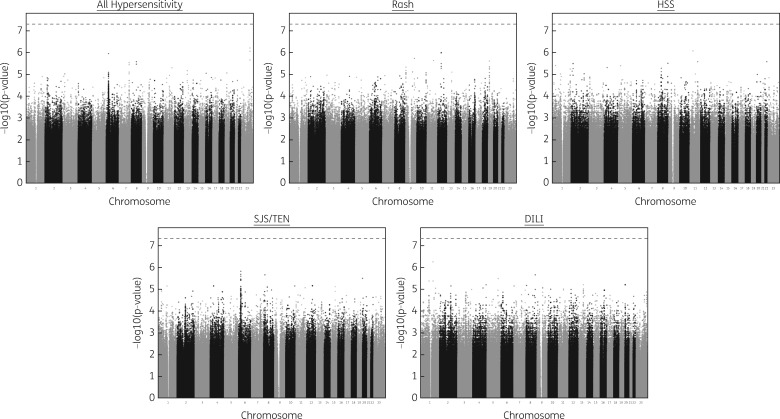
Manhattan plots of association for logistic regression SNP analysis for the five defined nevirapine hypersensitivity phenotypes. The phenotypes, all hypersensitivity, NIR and SJS/TEN show *P* values from logistic regression incorporating CD4+ count as a covariate. For HSS and DILI no covariates were incorporated. The broken line indicates genome-wide significance (*P *=* *5 × 10^−8^).

Univariate logistic regression analysis showed CD4+ cell count to be a statistically significant variable for NIR (*P *=* *0.016), SJS (*P *=* *0.003) and all hypersensitivity cases (*P *=* *2.5 × 10^−^^6^). Therefore, we included CD4+ cell count as a covariate in the SNP logistic regression model for these three phenotypes. Multidimensional scaling (MDS) variables were not included as covariates in the logistic regression since the population stratification analysis suggested that the cohort was homogeneous and genomic control was unnecessary (Figure S1). From the genome-wide logistic regression analyses, we identified 15 SNPs with *P *<* *1 × 10^−^^5^, with at least one of the five different phenotypes analysed (Figure 2; summarized in Table 2). No variant reached genome-wide significance.

**Table 2:** Top SNPs identified in association with a nevirapine hypersensitivity phenotype within the main cohort analysis

Phenotype	SNP	Chr	Position (GRCh37 .p13)	Reference allele	Associated allele	Gene	Typed/ imputed	Logistic regression	
								*P* value	OR (95% CI)
All hypersensitivity (*n* = 151)	rs34213790	6	31318579	G	A	3′ of *HLA-B*	imputed	1.15 × 10^−6^	2.34 (1.66–3.30)
	rs11764223	7	137742056	T	G	*AKR1D1*	imputed	2.99 × 10^−6^	0.45 (0.33–0.63)
	rs11988543	8	68596362	T	A	*CPA6*	imputed	2.69 × 10^−6^	3.37 (2.03–5.60)
	rs9527426	13	56208216	T	C	*MIR5007*	typed	9.86 × 10^−6^	2.25 (1.58–3.21)
	rs35990155	23	11827017	G	T	*KIAA1210*	imputed	6.33 × 10^−7^	5.65 (2.86–11.17)
Rash (*n* = 56)	rs10815440	9	661057	G	A	*KANK1*	typed	8.86 × 10^−6^	5.80 (2.68–12.67)
	rs115848367	10	128045499	A	C	*ADAM12*	imputed	8.83 × 10^−7^	4.70 (2.38–9.30)
	rs74373347	12	48919428	A	G	*OR8S1*	imputed	1.08 × 10^−6^	11.05 (4.12–29.04)
	rs6511720	19	11202306	G	T	*LDLR*	typed	5.49 × 10^−6^	4.66 (2.40–9.05)
SJS/TEN (*n* = 51)	rs5010528	6	31241032	A	G	*HLA-C*	typed	4.13 × 10^−6^	4.75 (2.45–9.22)
	rs150223496	15	76421885	G	A	*C15orf27*	imputed	8.20 × 10^−6^	17.34 (4.95–60.74)
DILI (*n* = 21)	rs147773805	1	107741393	A	T	*NTNG1*	imputed	4.47 × 10^−6^	11.05 (3.96–30.83)
	rs114693001	9	76074363	A	T	intergenic	imputed	8.82 × 10^−6^	9.35 (3.49–25.06)
	rs142213069	13	77962182	G	C	5′ of *MYCBP2*	imputed	6.64 × 10^−6^	14.42 (4.51–46.04)
	rs6139258	20	3958616	T	C	*RNF24*	typed	6.64 × 10^−6^	14.42 (4.52–46.04)

*P* values and ORs were determined by logistic regression with CD4+ cell count as a covariate (except for HSS and DILI).

### Replication cohort

Of the 15 SNPs considered for replication, one (rs150223496) could not be typed due to proximal sequence constraints of the Sequenom assay design process, and QC failure for TaqMan genotyping Hardy-Weinberg equilibrium (HWE) *P* value >0.0001, call rate >90%. Thus, 14 SNPs were carried forward for analysis. A total of 59 Malawian samples (30 cases and 29 controls) and 63 Ugandan samples (32 cases and 31 controls) passed QC (call rate >90%) as described in Table 1. Due to sample constraints, SNP signals identified in the discovery cohort for the ‘all hypersensitivity’ phenotype were not typed in the samples from Mozambique in the replication cohort.

For nevirapine-induced DILI (Table 3), combining the discovery and replication cohorts for the SNP rs6139258 in the *RNF24* locus strengthened the association [*P *=* *5.7 × 10^−^^7^, OR 13.62 (95% CI 4.90–37.84)] (Table 4). A single SNP (rs5010528 in the *HLA-C* locus) showed an association with SJS/TEN in the replication cohort (38 cases, 59 controls) [*P *=* *0.006; OR 5.12 (95% CI 1.60–16.42)]. Combining the discovery and replication cohorts strengthened the overall association, which approached genome-wide significance [*P *=* *8.5 × 10^−^^8^, OR 4.84 (95% CI 2.71–8.61)]. No positive signals were identified for the ‘all hypersensitivity’ phenotype (62 cases, 59 controls).

**Table 3:** Logistic regression analysis of candidate SNPs in the nevirapine hypersensitivity replication cohort

Phenotype	SNP	Chr	Position (GRCh37 .p13)	Genotyping platform	Reference allele	Associated allele	Gene	Logistic regression	
								*P* value	OR (95% CI)
All hypersensitivity (*n *= 62)	rs34213790	6	31318579	iPLEX	G	A	3′ of *HLA-B*	0.10	1.56 (0.94–2.60)
	^a^rs12112517	7	137743167	iPLEX	A	C	*AKR1D1*	0.21	1.42 (0.82–2.46)
	rs11988543	8	68596362	TaqMan	T	A	*CPA6*	0.77	1.13 (0.54–2.28)
	rs9527426	13	56208216	iPLEX	T	C	*MIR5007*	0.83	1.06 (0.62–1.80)
	rs35990155	23	11827017	iPLEX	G	T	*KIAA1210*	0.86	0.94 (0.48–1.86)
Rash (*n *= 27)	rs10815440	9	661057	iPLEX	G	A	*KANK1*	0.99	3.3 × 10^−10^ (0–∞)
	rs115848367	10	128045499	iPLEX	A	C	*ADAM12*	0.70	1.18 (0.51–2.70)
	rs74373347	12	48919428	iPLEX	A	G	*OR8S1*	0.76	0.81 (0.21–3.11)
	rs6511720	19	11202306	iPLEX	G	T	*LDLR*	0.86	1.07 (0.51–2.24)
SJS/TEN (*n *= 38)	rs5010528	6	31241032	TaqMan	A	G	*HLA-C*	**0.02**	5.33 (1.37–20.80)
	^b^rs150223496	15	76421885	NA	G	A	*C15orf27*	NA	NA
DILI (*n *= 42)	rs1730858	1	107619244	iPLEX	G	A	*NTNG1*	0.61	1.57 (0.27–8.97)
	^c^rs114693001	9	76074363	TaqMan	A	T	intergenic	0.99	2.6 × 10^−9^ (0–∞)
	^c^rs142213069	13	77962182	iPLEX	G	C	5′ of *MYCBP2*	0.99	2.4 × 10^−9^ (0–∞)
	rs6139258	20	3958616	iPLEX	T	C	*RNF24*	**0.03**	11.17 (1.29–96.38)

SNP associations below arbitrary statistical significance (*P *<* *0.1) are highlighted in bold. NA denotes statistical analysis not applicable.

a SNPs within the association signal that are substituted from the discovery cohort SNP (high LD) due to genotyping assay design constraints.

b SNP signal where replication in the replication cohort was not possible.

c SNPs that were not be typed in the Mozambique DILI cohort (*n *=* *15 cases). Mozambique individuals were also omitted from the ‘all hypersensitivity’ analysis.

**Table 4:** Meta-analysis of significantly associated SNPs in the discovery and replication cohorts

SNP	Gene	Phenotype	Cohort	Case/ control	*n*	MAF	Logistic regression		Meta-analysis	
							*P*	OR (95% CI)	*P*	OR (95% CI)
rs34213790	3′ of *HLA-B*	all hypersensitivity	discovery	case	151	0.45	1.15 × 10^−6^	2.34 (1.66–3.30)	–	–
				control	182	0.37				
			replication	case	27	0.50	0.10	1.56 (0.94–2.60)	6.69 × 10^−7^	2.06 (1.55–2.74)
				control	60	0.38				
rs5010528	*HLA-C*	SJS/TEN	discovery	case	51	0.36	4.13 × 10^−6^	4.75 (2.45–9.22)	–	–
				control	182	0.14				
			replication	case	38	0.38	6.03 × 10^−3^	5.12 (1.60–16.41)	8.47 × 10^−8^	4.84 (2.71–8.61)
				control	68	0.12				
rs6139258	*RNF24*	DILI	discovery	case	21	0.19	6.64 × 10^−6^	14.42 (4.52–46.04)	–	–
				control	182	0.02				
			replication	case	42	0.07	0.03	11.17 (1.29–96.38)	5.66 × 10^−7^	13.62 (4.90–37.84)
				control	68	0.01				

Data shown are for analysis undertaken with covariates (CD4+ cell count) included in the regression model (except DILI) for both the discovery and replication cohorts (± supplementary cohort for SJS/TEN and DILI).

### HLA-C allelotype imputation

Overall allelotype imputation from the SNP array data using HLA*IMP demonstrated 71.5% concordance with the HLA typing obtained for 116 of our patients using the sequence-based methodology. However the ability of the imputation to correctly call HLA-C*04:01 alleles was 90%.

Within the 116 patients for which HLA allelotyping and SNP array genotype data were available, *HLA-C-04:01* allele carriage co-occurred with the rs5010528 G allele in 112/116 cases (96.5%). For the imputed *HLA* allelotype data, C*04:01 co-occurred with rs5010528 G in 303/333 cases (91%).

**Figure 3:**
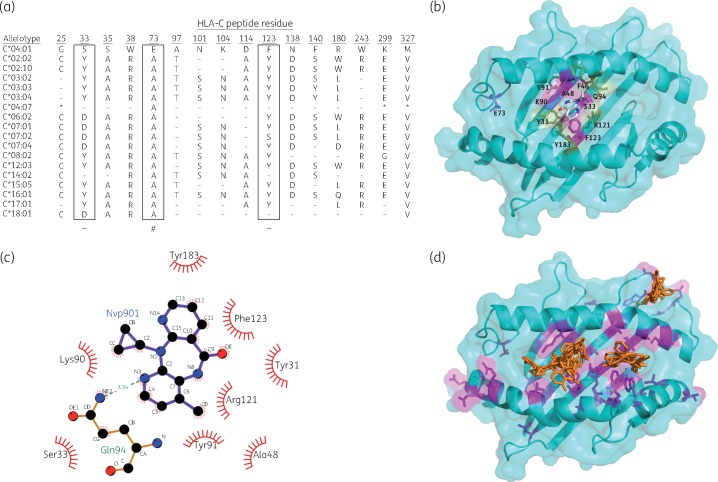
*In silico* docking of HLA-C*04:01 and nevirapine. (a) Specific peptide residues present in the different HLA-C allelotypes previously identified in the Malawian cohort (*n *=* *116). – shows continuity with the reference peptide (C*04:01) and * identifies peptides not sequenced in an allelotypes. ∼ denotes putative nevirapine interaction and # denotes residue substituted by SNP (rs1050409) identified in GWAS. Residue reference numbers are as defined by the *in silico* model. (b) HLA-C*04:01/nevirapine docking mode of conformation with the highest predicted affinity (lowest score) as produced using the PyMOL software. Key interacting residues are highlighted. (c) A LIGPLOT+^40^ 2D schematic representation of the interaction of nevirapine with HLA-C*04:01 PBD residues in the highest affinity predicted docking conformation mode. The broken line indicates a hydrogen bond and radiating lines indicate hydrophobic interactions. (d) HLA-C*04:01/nevirapine (orange) docking mode of conformation for the 20 highest predicted affinity (lowest scores) as produced using the PyMOL software.

The initial discovery logistic regression analysis demonstrated two non-synonymous SNPs in the *HLA-C* locus associated with SJS/TEN that were in absolute linkage disequilibrium (LD) with rs5010528 (Table 5). The first SNP (rs146911342) encodes a valine-to-methionine amino acid substitution at residue 327 and the second (rs1050409) encodes an alanine to glutamic acid at residue 73 (close to the peptide binding domain), which was also associated with SJS/TEN [*P *=* *4.1 × 10^−^^6^, OR 4.75 (95% CI2.45–9.23)]. Both are key defining residues of the HLA-C*04 allelotypes as defined in the HLA-IMGT database^34^ and within the Malawian cohort (Figure 3). Verification of either SNP in our discovery or replication cohorts via other genotyping methodologies was not possible due to sequence constraints in assay design (the proximal nucleotide sequence for primer design was not sufficiently unique or contained a restrictive number of other genetic variants).

**Table 5:** Association of nevirapine-induced SJS/TEN and imputed SNPs of the *HLA-C* locus from the main cohort and the targeted sequencing cohort

SNP	bp (GRCh37.p13)	A_1_/A_2_	Amino acid substitution	GWAS (discovery cohort)					Targeted sequencing cohort				
				typed/imputed	SJS/TEN MAF (*n*=51)	control MAF (*n*=182)	*P*	OR (95% CI)	SJS/TEN MAF (*n *= 16)	control MAF (*n *= 16)	*P*	OR (95% CI)	LD with rs5010528 (*D*′)
rs146911342	31 237 779	C/T	p.V327M	imputed	0.36	0.15	4.13 × 10^−6^	4.75 (2.45–9.23)	0.34	0.13	0.013	34.14 (2.07–561.6)	1
rs41562714	31 238 538	G/C		imputed	0.36	0.15	4.13 × 10^−6^	4.75 (2.45–9.23)	0.34	0.13	0.013	34.14 (2.07–561.6)	1
rs1050409	31 239 501	G/T	p.A73E	imputed	0.36	0.15	4.13 × 10^−6^	4.75 (2.45–9.23)	0.34	0.13	0.013	34.14 (2.07–561.6)	1
rs41553018	31 239 742	G/C		imputed	0.36	0.15	3.71 × 10^−6^	4.75 (2.45–9.23)	0.34	0.13	0.013	34.14 (2.07–561.6)	1
rs4361609	31 240 635	G/C		imputed	0.36	0.15	4.13 × 10^−6^	4.75 (2.45–9.23)	0.34	0.13	0.013	34.14 (2.07–561.6)	1
rs5010528	31 241 032	A/G		typed	0.36	0.15	4.13 × 10^−6^	4.75 (2.45–9.23)	0.34	0.13	0.013	34.14 (2.07–561.6)	–
rs58019823	31 241 215	A/G		imputed	0.36	0.15	4.13 × 10^−6^	4.75 (2.45–9.23)	–	–	–	–	NA
^a^rs2524087	31241 294	C/G		imputed	0.37	0.15	3.71 × 10^−6^	4.73 (2.45–9.14)	–	–	–	–	NA
^a^rs59103503	31 287 944	G/T		imputed	0.37	0.15	2.75 × 10^−6^	5.00 (2.55–9.80)	–	–	–	–	NA
^a^rs77641320	31 298 229	C/G		imputed	0.35	0.14	1.64 × 10^−6^	5.53 (2.75–11.12)	–	–	–	–	NA
^a^rs9468965	31 300 247	T/A		imputed	0.44	0.19	2.24 × 10^−6^	4.61 (2.24–8.69)	–	–	–	–	NA
^a^rs35364987	31 309 423	T/C		imputed	0.49	0.27	8.01 × 10^−6^	3.76 (2.10–6.74)	–	–	–	–	NA
^a^rs35435945	31 311 374	T/G		imputed	0.49	0.27	9.83 × 10^−6^	3.72 (2.08–6.67)	–	–	–	–	NA
^a^rs35278939	31 319 780	G/A		imputed	0.47	0.23	8.01 × 10^−6^	4.04 (2.19–7.47)	–	–	–	–	NA

The list comprises all SNPs with a *P* value of <1  × 10 ^−^ ^5^ in the main cohort analysis. Statistical significance and OR (95% CI) for the targeted sequencing cohort is determined by logistic regression with CD4+  cell count as covariate. NA denotes LD indeterminable in targeted sequencing cohort as SNP typing not available.

a SNP was imputed in the discovery cohort analysis but not detected and or called in the sequencing data.

However, targeted sequencing of the HLA locus in 16 SJS/TEN and 16 tolerant controls (Table 5) suggested that both the imputed non-synonymous SNPs may be in absolute LD with the original discovery cohort signal SNP (rs5010528), again demonstrating a significant association with nevirapine-induced SJS/TEN. Allelotype inference from the targeted sequencing SNP data also confirmed that both non-synonymous SNPs were in 100% co-occurrence with HLA-C*04:01 (data not shown). Our data do not show an association between any of the other *HLA* gene loci and other nevirapine-induced hypersensitivity phenotypes.

### HLA-C*04:01 and ERAP1 and ERAP2 SNP interactions

Given the previously reported interactions between ERAP genes and HLA class I-mediated diseases, in particular ankylosing spondylitis,^35^ we determined whether there was an interaction with the carriage of HLA-C*04:01 using the rs5010528 G allele as a proxy SNP (Table S1). There was no significant association (*P *>* *0.05) between the *ERAP1* variants and SJS/TEN risk in carriers of HLA-C*04:01. However, both *ERAP2* variants showed a nominal association with SJS/TEN risk [*P *=* *0.019, OR 0.43 (95% CI 0.21–0.87)], though this did not pass the Bonferroni threshold for multiple testing (*P *=* *0.0125).

### In silico docking

In light of the association observed between nevirapine-induced SJS/TEN and an SNP (rs1050409) encoding an amino acid substitution at residue 73 of HLA-C (p.A73E), *in silico* docking was undertaken to determine the possible effect of the residue substitution on nevirapine binding. The data suggest that none of the predicted modes of nevirapine docking conformation interact with residue 73, which appears to be on the periphery of the peptide-binding domain (Figure 3). The lowest scoring (predicted highest affinity) mode highlights an interaction between nevirapine and residues 33 and 123 in the B pocket (Figure 3). In HLA-C*04:01, residues 33 and 123 are serine and phenylalanine respectively (Figure 3). The majority of other allelotypes do not possess these particular residues (with the exception of C*04:07 and C*14:02). Docking of the metabolite 12-hydroxy-nevirapine was also undertaken, since it has also been suggested as potentially responsible for nevirapine-induced adverse drug reactions;^36^ these were in general agreement with those for nevirapine, in that docking seems to take place around the B pocket (e.g. near residues 33 and 123), but with more variability in the different modes predicted than for nevirapine. None of the predicted modes interacted with residue 73 (data not shown). Taken together, the docking results suggest that binding of either nevirapine or 12-hydroxy-nevirapine around the centre of the peptide-binding regions is likely to be important in the mechanism of the immune-mediated reaction.

## Discussion

The investigation of genetic factors predisposing to serious adverse drug reactions is challenging because of their rarity. Despite this, we have assembled one of the largest cohorts of patients with clinically well-characterized nevirapine hypersensitivity, including SJS/TEN. GWAS analysis of our Malawian discovery cohort (*n *=* *333) identified 15 polymorphisms having a suggestive association with nevirapine hypersensitivity (Table 2). Subsequent analysis of these variants in our replication cohort suggested that three of the SNPs may be potential risk factors (Table 3): rs34213790 3′ of the *HLA-B* gene locus with all hypersensitivity phenotypes; rs5010528 in the *HLA-C* gene locus with SJS/TEN, and rs6139258 in the *RNF24* gene locus with DILI. The weakest of the above three association signals, SNP rs34213790, is unlikely to be an independent marker of nevirapine hypersensitivity in general, and its association may be due to a haplotype effect between HLA-C*04:01 (rs5010528; see below) and B allelotypes.

SNP rs6139258 in the *RNF24* gene locus only marginally failed to pass the Bonferroni threshold of significance in the replication cohort. Very little is known regarding the function of *RNF24*. However, it is known that it is a protein that interacts with transient receptor potential cation channel 6 (TRPC6),^37^ a receptor-activated channel, expressed in liver cells,^38^ which plays a role in cellular calcium homeostasis. *TRPC6* has been suggested to play a role in hepatoma cell-line proliferation, possibly via a cyclin D-modulated mechanism.^38^ Thus *RNF24* may have some biological plausibility in the pathogenesis of nevirapine-induced liver injury, and merits further investigation in additional patients with nevirapine-induced DILI, and functional work to uncover the possible mechanisms (if any) of the association.

The most compelling of the three signals, rs5010528, gave an OR of 4.84 for nevirapine-induced SJS/TEN, and was replicated in patients from three countries (Malawi, Uganda and Mozambique) at the Bonferroni threshold (*P *<* *0.05), approaching genome-wide significance in the combined analysis (*P *=* *8.5 × 10^−^^8^) (Table 4). SNP rs5010528 is located within the *HLA-C* gene locus. High co-occurrence of rs5010528 with HLA-C*04:01 was observed (96.5%) in 116 patients within this study who had previously been HLA typed by sequence-based methods. The association between nevirapine and rs5010528 (as a proxy for C*04:01) can be considered statistically significant when applying an HLA-wide significance threshold of *P *<* *2.5 × 10^−^^4^. Additionally, *HLA-C* allelotypes imputed from the SNP array data also showed a high co-occurrence (91%) in the main study cohort, suggesting rs5010528 may be a good proxy for *HLA*-C*04:01. Thus, the GWAS data appear to confirm our previous finding^21^ associating HLA-C*04:01 with nevirapine-induced SJS/TEN. Of note, the association of rs5010528 with other hypersensitivity phenotypes was not as strong, suggesting that the risk conferred by rs5010528 and thus *HLA*-C*04:01 is specific for nevirapine-induced SJS/TEN. The reason for this is unclear, and will require further investigation.

In terms of clinical utility, rs5010528 appears to have little potential as a pre-emptive genetic test. Indeed, based on a prevalence of SJS/TEN in our prospective cohort of 1.07% and assuming a dominant mode of inheritance, the positive (PPV) and negative (NPV) predictive values were 2.8% and 42.4% respectively. For the *RNF24* variant (rs6139258) the PPV, based on a prevalence of DILI of 0.63%, is also very low (0.2%).

Only one previous GWAS investigating nevirapine hypersensitivity has been reported, but in a smaller Thai population (72 cases, 77 controls).^39^ Patients had a wide variety of rashes, with only 11 grade 4 cases (6.9%), which would be equivalent to our cases with SJS/TEN. The SNP rs9461684 in the *HLA-C* locus was significantly associated with nevirapine rash, but no *HLA* allelotype imputation or *HLA* sequencing was carried out. In our data, rs9461684 is in high LD with our top SNP, rs5010528 (*D*′ = 1.0, *r*^2^ = 0.972). The discrepancy may be a result of the different LD patterns in the different ethnic groups studied, as well as the much lower numbers of patients with serious skin reactions in the Thai study.

From the imputed SNP data of the discovery cohort and targeted resequencing data (Table 5), it is clear that rs5010528 is in LD with a functional non-synonymous SNP (rs1050409) that leads to an alanine-to-glutamic acid substitution at residue 73, which lies near to the peptide-binding domain of the HLA-C protein. However, *in silico* modelling suggested that this residue does not interact with nevirapine in any docking conformation. Two other residues of HLA-C*04:01 (33 and 123 in the model) appear to be the key interactors in the majority of the predicted modes of nevirapine docking (Figure 3), including the most favoured. However, it should be noted that this is a predictive model and further analysis of the HLA-C/nevirapine complex is needed to further elucidate the potential for docking. The association signal at residue 73 (rs1050409) is likely to be a proxy for the 33 and 123 residues also present in HLA-C*04:01. However, this work has provided the first evidence that nevirapine binds to the B pocket of HLA-C*04:01.

*ERAP* gene variants interact in a protective manner in HLA-mediated diseases such as ankylosing spondylitis in individuals who carry the risk *HLA* alleles.^35^ ERAP1 and ERAP2 are enzymes involved in antigenic peptide precursor trimming prior to loading into HLA class I molecules (and may thus alter the peptidome) and may potentially also alter the expression of the risk HLA class I allele. To our knowledge, this is one of the first examinations of whether there is interaction between drug-induced HLA disease and the *ERAP* genes. We were, however, unable to detect an interaction between *ERAP1* variants and HLA-C*04:01 in African patients with SJS/TEN. However, a nominal association (*P *=* *0.019) was observed for both *ERAP2* SNPs (Table S1). A limitation of our analysis is the small sample size, particularly given the much larger numbers that have been studied in ankylosing spondylitis. Nevertheless, the possibility of an association with ERAP2 is intriguing, and needs further investigation not only with nevirapine-induced hypersensitivity, but also with other HLA-related adverse drug reactions.

In identifying an SNP in the *HLA-C* locus that appears to be a proxy for the HLA-C*04:01 allele, as a risk factor for nevirapine-induced SJS/TEN, this study has added further weight to existing evidence. The data generated also suggest that, in sub-Saharan African HIV patients, no other strong, significant genetic risk factors for nevirapine hypersensitivity exist that could be utilized as clinical predictive markers. However, the data are valuable in terms of the mechanistic insights they provide. Additionally, *in silico* analysis has identified two putative HLA-C peptide residues that are predicted to be key for the binding of nevirapine, which warrant further investigation as to their role in the pathogenesis of SJS/TEN. Further work is also needed to determine the reasons for organ-specific toxicities in different patients.

## Supplementary Material

Supplementary Data

## References

[1] van Leth F, Phanuphak P, Ruxrungtham K Comparison of first-line antiretroviral therapy with regimens including nevirapine, efavirenz, or both drugs, plus stavudine and lamivudine: a randomised open-label trial, the 2NN Study. Lancet 2004; 363: 1253–63. 15094269 10.1016/S0140-6736(04)15997-7

[2] Siegfried NL, Van Deventer PJ, Mahomed FA Stavudine, lamivudine and nevirapine combination therapy for treatment of HIV infection and AIDS in adults. Cochrane Database Syst Rev 2006; 19: issue CD004535. 10.1002/14651858.CD004535.pub2 PMC8407055 16625606

[3] Phillips E, Gutierrez S, Jahnke N Determinants of nevirapine hypersensitivity and its effect on the association between hepatitis C status and mortality in antiretroviral drug-naive HIV-positive patients. AIDS 2007; 21: 1561–8. 17630551 10.1097/QAD.0b013e3282170a9d

[4] Wit FW, Kesselring AM, Gras L Discontinuation of nevirapine because of hypersensitivity reactions in patients with prior treatment experience, compared with treatment-naive patients: the ATHENA cohort study. Clin Infect Dis 2008; 46: 933–40. 18271750 10.1086/528861

[5] Fagot JP, Mockenhaupt M, Bouwes-Bavinck JN Nevirapine and the risk of Stevens-Johnson syndrome or toxic epidermal necrolysis. AIDS 2001; 15: 1843–8. 11579247 10.1097/00002030-200109280-00014

[6] Mittmann N, Knowles SR, Koo M Incidence of toxic epidermal necrolysis and Stevens-Johnson syndrome in an HIV cohort: an observational, retrospective case series study. Am J Clin Dermatol 2012; 13: 49–54. 22145749 10.2165/11593240-000000000-00000

[7] De Maat MM, Mathot RA, Veldkamp AI Hepatotoxicity following nevirapine-containing regimens in HIV-1-infected individuals. Pharmacol Res 2002; 46: 295–300. 12220974 10.1016/s1043-6618(02)00146-9

[8] Riska P, Lamson M, MacGregor T Disposition and biotransformation of the antiretroviral drug nevirapine in humans. Drug Metab Dispos 1999; 27: 895–901. 10421616

[9] Jinno H, Tanaka-Kagawa T, Ohno A Functional characterization of cytochrome P450 2B6 allelic variants. Drug Metab Dispos 2003; 31: 398–403. 12642465 10.1124/dmd.31.4.398

[10] Lang T, Klein K, Fischer J Extensive genetic polymorphism in the human CYP2B6 gene with impact on expression and function in human liver. Pharmacogenetics 2001; 11: 399–415. 11470993 10.1097/00008571-200107000-00004

[11] Rotger M, Colombo S, Furrer H Influence of CYP2B6 polymorphism on plasma and intracellular concentrations and toxicity of efavirenz and nevirapine in HIV-infected patients. Pharmacogenet Genomics 2005; 15: 1–5. 15864119 10.1097/01213011-200501000-00001

[12] Penzak SR, Kabuye G, Mugyenyi P Cytochrome P450 2B6 (CYP2B6) G516T influences nevirapine plasma concentrations in HIV-infected patients in Uganda. HIV Med 2007; 8: 86–91. 17352764 10.1111/j.1468-1293.2007.00432.x

[13] Yuan J, Guo S, Hall D Toxicogenomics of nevirapine-associated cutaneous and hepatic adverse events among populations of African, Asian, and European descent. AIDS 2011; 25: 1271–80. 21505298 10.1097/QAD.0b013e32834779df PMC3387531

[14] Haas DW, Bartlett JA, Andersen JW Pharmacogenetics of nevirapine-associated hepatotoxicity: an Adult AIDS Clinical Trials Group collaboration. Clin Infect Dis 2006; 43: 783. 16912957 10.1086/507097

[15] Martin AM, Nolan D, James I Predisposition to nevirapine hypersensitivity associated with HLA-DRB1*0101 and abrogated by low CD4 T-cell counts. AIDS 2005; 19: 97–9. 15627041 10.1097/00002030-200501030-00014

[16] Vitezica ZG, Milpied B, Lonjou C HLA-DRB1*01 associated with cutaneous hypersensitivity induced by nevirapine and efavirenz. AIDS 2008; 22: 540–1. 18301070 10.1097/QAD.0b013e3282f37812

[17] Likanonsakul S, Rattanatham T, Feangvad S HLA-Cw*04 allele associated with nevirapine-induced rash in HIV-infected Thai patients. AIDS Res Ther 2009; 6: 22. 19845952 10.1186/1742-6405-6-22 PMC2774340

[18] Gao S, Gui XE, Liang K HLA-dependent hypersensitivity reaction to nevirapine in Chinese Han HIV-infected patients. AIDS Res Hum Retroviruses 2011; 28: 540–3. 21902584 10.1089/AID.2011.0107

[19] Gatanaga H, Yazaki H, Tanuma J HLA-Cw8 primarily associated with hypersensitivity to nevirapine. AIDS 2007; 21: 264–5. 17197830 10.1097/QAD.0b013e32801199d9

[20] Chantarangsu S, Mushiroda T, Mahasirimongkol S HLA-B*3505 allele is a strong predictor for nevirapine-induced skin adverse drug reactions in HIV-infected Thai patients. Pharmacogenet Genomics 2009; 19: 139–46. 19104471 10.1097/FPC.0b013e32831d0faf

[21] Carr DF, Chaponda M, Jorgensen AL Association of human leukocyte antigen alleles and nevirapine hypersensitivity in a Malawian HIV-infected population. Clin Infect Dis 2013; 56: 1330. 23362284 10.1093/cid/cit021 PMC3616517

[22] Kenna TJ, Robinson PC, Haroon N. Endoplasmic reticulum aminopeptidases in the pathogenesis of ankylosing spondylitis. Rheumatology (Oxford) 2015; 54: 1549–56. 26070942 10.1093/rheumatology/kev218

[23] Mugyenyi P, Walker AS, Hakim J Routine versus clinically driven laboratory monitoring of HIV antiretroviral therapy in Africa (DART): a randomised non-inferiority trial. Lancet 2010; 375: 123–31. 20004464 10.1016/S0140-6736(09)62067-5 PMC2805723

[24] Ciccacci C, Borgiani P, Ceffa S Nevirapine-induced hepatotoxicity and pharmacogenetics: a retrospective study in a population from Mozambique. Pharmacogenomics 2010; 11: 23–31. 20017669 10.2217/pgs.09.142

[25] Ciccacci C, Di Fusco D, Marazzi MC Association between CYP2B6 polymorphisms and nevirapine-induced SJS/TEN: a pharmacogenetics study. Eur J Clin Pharmacol 2013; 69: 1909–16. 23774940 10.1007/s00228-013-1549-x

[26] Munderi P, Snowden WB, Walker AS Distribution of HLA-B alleles in a Ugandan HIV-infected adult population: NORA pharmacogenetic substudy of DART. Trop Med Int Health 2011; 16: 200–4. 21091860 10.1111/j.1365-3156.2010.02688.x

[27] Purcell S, Neale B, Todd-Brown K PLINK: a tool set for whole-genome association and population-based linkage analyses. Am J Hum Genet 2007; 81: 559–75. 17701901 10.1086/519795 PMC1950838

[28] Delaneau O, Zagury JF, Marchini J. Improved whole-chromosome phasing for disease and population genetic studies. Nat Methods 2013; 10: 5–6. 23269371 10.1038/nmeth.2307

[29] Howie BN, Donnelly P, Marchini J. A flexible and accurate genotype imputation method for the next generation of genome-wide association studies. PLoS Genet 2009; 5: e1000529. 19543373 10.1371/journal.pgen.1000529 PMC2689936

[30] Marchini J, Howie B. Genotype imputation for genome-wide association studies. Nat Rev Genet 2010; 11: 499–511. 20517342 10.1038/nrg2796

[31] R Core Team (2013). R: A Language and Environment for Statistical Computing. Vienna, Austria: R Foundation for Statistical Computing ISBN 3-900051-07-0. http://www.R-project.org.

[32] Magi R, Morris AP. GWAMA: software for genome-wide association meta-analysis. BMC Bioinformatics 2010; 11: 288. 20509871 10.1186/1471-2105-11-288 PMC2893603

[33] Dilthey A, Leslie S, Moutsianas L Multi-population classical HLA type imputation. PLoS Comput Biol 2013; 9: e1002877. 23459081 10.1371/journal.pcbi.1002877 PMC3572961

[34] Robinson J, Halliwell JA, Hayhurst JD The IPD and IMGT/HLA database: allele variant databases. Nucleic Acids Res 2015; 43: D423–31. 25414341 10.1093/nar/gku1161 PMC4383959

[35] Evans DM, Spencer CC, Pointon JJ Interaction between ERAP1 and HLA-B27 in ankylosing spondylitis implicates peptide handling in the mechanism for HLA-B27 in disease susceptibility. Nat Genet 2011; 43: 761–7. 21743469 10.1038/ng.873 PMC3640413

[36] Sharma AM, Novalen M, Tanino T 12-OH-nevirapine sulfate, formed in the skin, is responsible for nevirapine-induced skin rash. Chem Res Toxicol 2013; 26: 817–27. 23590230 10.1021/tx400098z

[37] Lussier MP, Lepage PK, Bousquet SM RNF24, a new TRPC interacting protein, causes the intracellular retention of TRPC. Cell Calcium 2008; 43: 432–43. 17850865 10.1016/j.ceca.2007.07.009

[38] El Boustany C, Bidaux G, Enfissi A Capacitative calcium entry and transient receptor potential canonical 6 expression control human hepatoma cell proliferation. Hepatology 2008; 47: 2068–77. 18506892 10.1002/hep.22263

[39] Chantarangsu S, Mushiroda T, Mahasirimongkol S Genome-wide association study identifies variations in 6p21.3 associated with nevirapine-induced rash. Clin Infect Dis 2011; 53: 341–8. 21810746 10.1093/cid/cir403

[40] Laskowski RA, Swindells MB. LigPlot+: multiple ligand-protein interaction diagrams for drug discovery. J Chem Inf Model 2011; 51: 2778–86. 21919503 10.1021/ci200227u

