# Supplementary material for: Genome-wide association study of nevirapine hypersensitivity in a sub-Saharan African HIV-infected population
Source: J Antimicrob Chemother. 2017 Jan 5;72(4):1152–62. doi: 10.1093/jac/dkw545 (PMC5400091; doi:10.1093/jac/dkw545)
Supplement: Supplementary Data [file dkw545_Supp.docx]

**SUPPLEMENTARY DATA**

**METHODS**

***In silico* docking**

The *HLA-C*04:01* crystal structure was retrieved from the Protein Data Bank[^1^](#_ENREF_1) (www. rcsb.org) (accession 1QQD [^2^](#_ENREF_2)). Protein structures of other HLA-C alleles were modelled using the Relax protocol of Rosetta 2015.05 ^[3](#_ENREF_3)^, with 1QQD as a template. Generation of PDBQT files for both receptor and ligand and docking were performed using AutoDock Tools and Autodock VINA respectively [^4^](#_ENREF_4), using a grid of 42 x 50 x 50 Å and exhaustiveness = 30. Five replicates of docking were performed, with each generating 20 docking poses. There was little difference between poses of each replicate, and so the first replicate was utilised. Affinity scores were estimated for each of the 20 docking poses. Lower scores indicate higher predicted affinity of protein for ligand. Graphical representations of the docking were produced using PyMOL ([www.pymol.org](http://www.pymol.org)).

**Targeted Sequencing of MHC region**

***Library preparation and Sequencing***

SureSelect^XT^ libraries were prepared and hybridised according to the SureSelect MHC Manual for longer insert size (version 6, 31.05.2011) (Agilent Technologies, Santa Clara, CA), using 3μg of genomic DNA (or all available) as input material. For amplification of pre-capture libraries, 250ng of adapter-ligated DNA was subjected to 5 cycles of PCR according to the manufacturer’s protocol. 630-750ng of pre-capture library was hybridised to 2μl of custom SureSelect RNA oligomer baits for approximately 24 hours at 65°C. DNA was retained on the streptavidin beads, which were re-suspended in 30μl of nuclease-free water, as per the updated standard SureSelect^XT^ for Illumina protocol (version 1.6, October 2013). Captured library was amplified and indexed on-bead using 12 PCR cycles. Final libraries were quantified by Qubit double-stranded DNA High Sensitivity assay (Life Technologies, Paisley, UK) and the size distribution determined by analysis on a Fragment Analyser using a High Sensitivity NGS Kit (both Advanced Analytical Technologies Inc, Ames, IA). Libraries (32 in total) were pooled in equimolar amounts based on the aforementioned Qubit and Fragment Analyser data. The pool was further purified using Axygen AxyPrep Mag PCR Clean-Up beads (Corning Inc., Corning, NY). The size of the final pool was assessed on a Bioanalyser High Sensitivity DNA chip (Agilent Technologies) and the DNA concentration was determined initially by Qubit double-stranded DNA High Sensitivity assay, and then by qPCR, using an Illumina Library Quantification Kit (KAPA) (Illumina Inc., San Diego CA) on a LightCycler 480 II system (Roche Diagnostics, Burgess Hill, UK). Sequencing was carried out on 1 lane of an HiSeq 2000 (Illumina) with version 3 chemistry generating 2 × 100bp paired end reads**.**

***Read Alignment and Variant Calling***

Non-informative duplicate reads, usually arising from PCR amplification or as optical duplicates during sequencing, were removed using the MarkDuplicates module of Picard (<http://broadinstitute.github.io/picard/>, version 1.94(1484)). Local realignment of reads around insertions and deletions was performed to reduce mismatching bases across all reads, with the IndelRealigner module of GATK version 2.6-4-g3e5ff60 [^5^](#_ENREF_5). SNP, insertion and deletion discovery was performed with the UnifiedGenotyper module of GATK version 2.6-4-g3e5ff60 ^[6](#_ENREF_6)^, restricted to the regions defined in S0404072_Covered.txt. During variant calling, variants were annotated using dbSNP v138. Detected variants were processed and filtered using the VariantFiltration module of GATK version 2.6-4-g3e5ff60 [^5^](#_ENREF_5), following best practices [^7^](#_ENREF_7).

**Imputation of HLA- allelotype and MHC locus**

Imputation of HLA-C allelotype from the discovery cohort SNP array data was undertaken using HLA*IMP:02 [^8^](#_ENREF_8) with a missing data threshold set at 0.20 and using the multi-ethnic training set. Concurrence of the imputation methodology was assessed by comparing predicted allelotype with allelotypes from 116 main cohort samples typed previously by sequencing based protocols ^[9](#_ENREF_9)^. Imputation of the MHC locus was also undertaken using pre-phased haplotypes from the discovery cohort up to the 1000 genomes phase 3 reference panel. This reference panel incorporates haplotypes from a wider range of African ancestry populations than phase 1, thus enabling improved imputation to lower allele frequencies in the discovery cohort. SNPs with an information measure (info score) below 0.8 were discarded. Association with hypersensitivity phenotypes was tested in a logistic regression model, incorporating SNP dosage to account for imputation uncertainty, after adjustment for the clinical covariates identified in discovery analyses, using SNPTEST ^[10](#_ENREF_10)^.

**RESULTS**


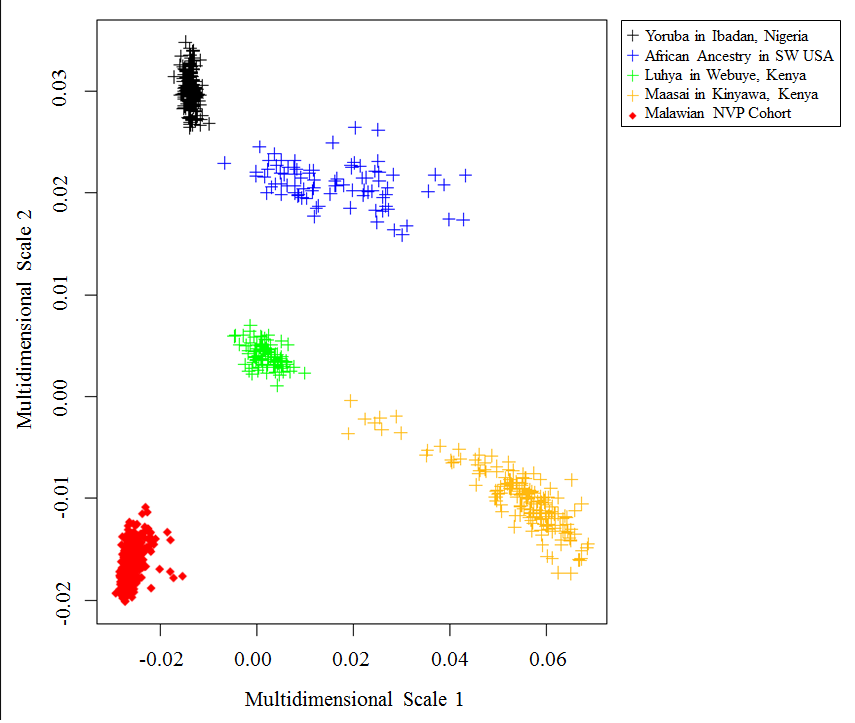


**Figure S1.** Multidimensional scaling (MDS) analysis for population stratification of the nevirapine discovery cohort with other sub-Saharan populations from HapMap phase 3. X and Y axis represent the 2 multi-dimensional scales as determined using PLINK. The nevirapine discovery cohort are represented by red diamonds and the HapMap 3 populations by the different colour crosses as indicated in the key.

**Table S1.** Association of *ERAP1* polymorphisms with nevirapine-induced SJS/TEN in HLA-C*04:01 positive individuals. *HLA-C*04:01* carriageis stated using rs5010528 as a presumptive proxy. P-value and odds ratio (with 95% CI) are determined by logistic regression with CD4+ cell count as a covariate.

|  |  |  |  |  | **SJS/TEN** | | | | **Tolerant** | | | |  | |
| --- | --- | --- | --- | --- | --- | --- | --- | --- | --- | --- | --- | --- | --- | --- |
|  |  |  |  |  | ***HLA-C*04:01* +ve (n=35)** | | | | ***HLA-C*04:01* +ve (n=48)** | | | | **Logistic Regression** | |
|  |  | **bp**  **(GRCh37.p13** | **AA Substitution** | **A1/A2** | **A1/A1** | **A1/A2** | **A2/A2** | **MAF** | **A1/A1** | **A1/A2** | **A2/A2** | **MAF** | **OR (95% CI)** | **p** |
| ***ERAP1*** | **rs30187** | chr5:96124330 | p.K528R | C/T | 9 | 18 | 8 | 0.49 | 10 | 25 | 13 | 0.47 | 1.31 (0.67-2.56) | 0.438 |
|  | **rs10050860** | chr:96122210 | p.D575N | C/T | 29 | 6 | 0 | 0.09 | 41 | 7 | 0 | 0.07 | 1.11(0.31-3.95) | 0.875 |
|  |  |  |  |  |  |  |  |  |  |  |  |  |  |  |
| ***ERAP2*** | **rs2549782** | chr5:96231000 | p.K392N | G/T | 12 | 18 | 5 | 0.40 | 11 | 20 | 17 | 0.56 | 0.43 (0.21-0.87) | 0.019 |
|  | **rs2248374** | chr5:96235896 | Intronic | A/G | 12 | 18 | 5 | 0.40 | 11 | 20 | 17 | 0.56 | 0.43 (0.21-0.87) | 0.019 |

**REFERENCES**

1. Berman HM, Westbrook J, Feng Z et al. The Protein Data Bank. *Nucleic Acids Res* 2000; **28**: 235-42.

2. Fan QR, Wiley DC. Structure of human histocompatibility leukocyte antigen (HLA)-Cw4, a ligand for the KIR2D natural killer cell inhibitory receptor. *J Exp Med* 1999; **190**: 113-23.

3. Leaver-Fay A, Tyka M, Lewis SM et al. ROSETTA3: an object-oriented software suite for the simulation and design of macromolecules. *Meth Enzymol* 2011; **487**: 545-74.

4. Morris GM, Huey R, Lindstrom W et al. AutoDock4 and AutoDockTools4: Automated docking with selective receptor flexibility. *J Comp Chem* 2009; **30**: 2785-91.

5. McKenna A, Hanna M, Banks E et al. The Genome Analysis Toolkit: a MapReduce framework for analyzing next-generation DNA sequencing data. *Genome research* 2010; **20**: 1297-303.

6. DePristo MA, Banks E, Poplin R et al. A framework for variation discovery and genotyping using next-generation DNA sequencing data. *Nature genetics* 2011; **43**: 491-8.

7. Van der Auwera GA, Carneiro MO, Hartl C et al. From FastQ data to high confidence variant calls: the Genome Analysis Toolkit best practices pipeline. *Curr Protoc Bioinformatics* 2013; **11**: 11 0 1- 0 33.

8. Dilthey A, Leslie S, Moutsianas L et al. Multi-population classical HLA type imputation. *PLoS Comput Biol* 2013; **9**: e1002877.

9. Carr DF, Chaponda M, Jorgensen AL et al. Association of human leukocyte antigen alleles and nevirapine hypersensitivity in a Malawian HIV-infected population. *Clin Infect Dis* 2013; **56**: 1330-9.

10. Marchini J, Howie B. Genotype imputation for genome-wide association studies. *Nat Rev Genet*2010; **11**: 499-511.
